# Metal–Organic Framework-Based Nanostructures for Electrochemical Sensing of Sweat Biomarkers

**DOI:** 10.3390/bios14100495

**Published:** 2024-10-12

**Authors:** Jing Meng, Moustafa Zahran, Xiaolin Li

**Affiliations:** 1 School of Civil Engineering, Nantong Institute of Technology, Nantong 226002, China; 2 Institute of Intelligent Manufacturing Technology, Shenzhen Polytechnic University, Shenzhen 518055, China

**Keywords:** sweat, metal–organic framework, nanoparticle, electrochemical sensor, wearable sensor

## Abstract

Sweat is considered the most promising candidate to replace conventional blood samples for noninvasive sensing. There are many tools and optical and electrochemical methods that can be used for detecting sweat biomarkers. Electrochemical methods are known for their simplicity and cost-effectiveness. However, they need to be optimized in terms of selectivity and catalytic activity. Therefore, electrode modifiers such as nanostructures and metal–organic frameworks (MOFs) or combinations of them were examined for boosting the performance of the electrochemical sensors. The MOF structures can be prepared by hydrothermal/solvothermal, sonochemical, microwave synthesis, mechanochemical, and electrochemical methods. Additionally, MOF nanostructures can be prepared by controlling the synthesis conditions or mixing bulk MOFs with nanoparticles (NPs). In this review, we spotlight the previously examined MOF-based nanostructures as well as promising ones for the electrochemical determination of sweat biomarkers. The presence of NPs strongly improves the electrical conductivity of MOF structures, which are known for their poor conductivity. Specifically, Cu-MOF and Co-MOF nanostructures were used for detecting sweat biomarkers with the lowest detection limits. Different electrochemical methods, such as amperometric, voltammetric, and photoelectrochemical, were used for monitoring the signal of sweat biomarkers. Overall, these materials are brilliant electrode modifiers for the determination of sweat biomarkers.

## 1. Introduction

Nanostructures have made significant contributions to various fields such as biotechnology, food, and medicine due to their outstanding chemical and physical characteristics [1,2]. Several substances such as metals, metal oxides, ceramics, and polymers can be used for the preparation of nanostructures [3]. Nanostructures, which have many unique properties, such as a high surface area to volume ratio and improved electronic and catalytic features, are considered promising materials for electrochemical sensors [4,5,6,7,8].

Electrochemical nanosensors have been utilized for detecting environmental pollutants and diagnosing diseases [9]. The incorporation of nanostructures into electrochemical sensors leads to an enhanced electrochemical signal with superior sensitivity and decreased detection limits [10,11]. Different nanostructures, such as metal nanoparticles (NPs), metal oxide NPs, carbon-based nanostructures, nano-polymers, molecularly imprinted polymers (MIP), and metal–organic framework (MOF)-based nanostructures, have been introduced into electrochemical sensors [12,13,14,15].

MOFs, organic–inorganic composites, were developed in the past decade. They are porous materials such as carbon nanotubes (CNTs) and zeolitic materials [16]. The MOF structure is composed of metal ions interacting with organic ligands. MOFs can be classified into many categories, such as Fe-MOF [17], Co-MOF [18], Cu-MOF [19], Ni-MOF [20], Zn-MOF [21], and Zr-MOF [22], based on the metal ion. Additionally, they can be classified into zeolitic, channel-type, or cage-type according to the pore geometry. Moreover, they can be grouped into zero-, one-, two-, and three-dimensional MOFs [23]. Generally, MOFs are utilized as templates for the design and fabrication of high-performance hollow nanostructures [24]. Additionally, MOF structures have been applied in gas storage, energy storage, separation, pollutant removal, catalysis, and sensing [25,26,27,28,29,30,31,32].

MOFs are gaining serious attention in the field of chemical sensing owing to the designability of metals and ligands, tunable pore size, and plentiful functional sites [33]. Previously, they were integrated with optical sensors such as colorimetric [34,35], fluorescence [36], and surface-enhanced Raman spectroscopy (SERS) [37] reagents to determine the analytes in environmental and biological samples. Recently, MOFs have been introduced as electrochemical probes for boosting sensitivity and selectivity as well as increasing the active surface area of the electrochemical sensors [38]. MOFs showed attractive characteristics such as a high porosity and increased surface area, which are beneficial for boosting the performance of electrochemical sensors [39,40]. Additionally, most MOF-modified electrodes have shown a high stability and good reproducibility of electrochemical responses, which makes them brilliant electrode modifiers for detecting many targeted analytes [41]. Reportedly, they have been used for detecting many environmental [42] and biological analytes [43]. However, the poor conductivity of many MOF structures, which resulted from the absence of low-energy paths for charge transfer and free-moving carriers, hinders its implementation in sensing devices [44]. Thus, the pyrolysis of MOFs, the optimization of metal-centered electronic structure, the synthesis of low-dimensional MOFs, or their combination with conductive materials have been used to deal with this problem [45].

Sweat biomarkers are crucial for monitoring health status and disease diagnosis. Liquid chromatography [46], ELISA [47], and optical immunosensors [48] were used for the determination of sweat biomarkers. However, these tools are complex, time-consuming, and expensive [49,50]. Therefore, a significant amount of focus has been put into electrochemical methods, especially those based on nanostructures, for enhancing the determination of sweat biomarkers. In this review, we focus on MOF-based nanostructures as conductive materials for enhancing the electrochemical determination of sweat biomarkers. MOF-based nanostructures are considered simple, sensitive, inexpensive, and effective electrochemical probes for determining sweat biomarkers compared with the other analytical methods.

## 2. Sweat Biomarkers

Sweating is a biological process for the detoxification and body’s thermoregulation [51]. Sweat is an attractive biofluid, which is secreted by sweat glands, for noninvasive biosensing due to its enrichment of metabolites, electrolytes, hormones, and proteins. These components are considered important signals for monitoring health status [52,53,54]. Specifically, sweat components including glucose, lactic acid, cortisol, uric acid, and ascorbic acid have been tested as biomarkers for many diseases. Glucose has a significant role in metabolic functions [55]. The determination of the concentration of sweat glucose is crucial for controlling glycometabolism-related diseases [56]. Lactate is used to evaluate a person’s physical performance. It is derived from glycogen stored in muscles for supplying energy to the body during exercise [30]. Additionally, the sharp increase in lactic acid in sweat causes stress ischemia [57]. Moreover, the acidity of lactic acid can lower blood pH, leading to a disruption in the body’s metabolic processes [58]. Cortisol, a stress hormone, is considered a reliable biomarker for evaluating human stress in addition to its role in assisting individuals in adaptation to various endogenous and exogenous stimuli. Additionally, it maintains immune function, energy metabolism, and electrolyte balance [31]. Uric acid, a metabolite of purine nucleotides in the human body, is relevant to cardiovascular and renal diseases [59]. Additionally, pH has been studied as a sweat biomarker for many diseases, such as Alzheimer’s disease, wound infection, renal failure, and cancer [60,61]. Moreover, vitamins [62], drugs [63,64], nicotine [65], and toxic metal ions [66] were efficiently studied as sweat biomarkers of significant interest.

## 3. Electrochemical Detection of Sweat Biomarkers

**Figure 1:**
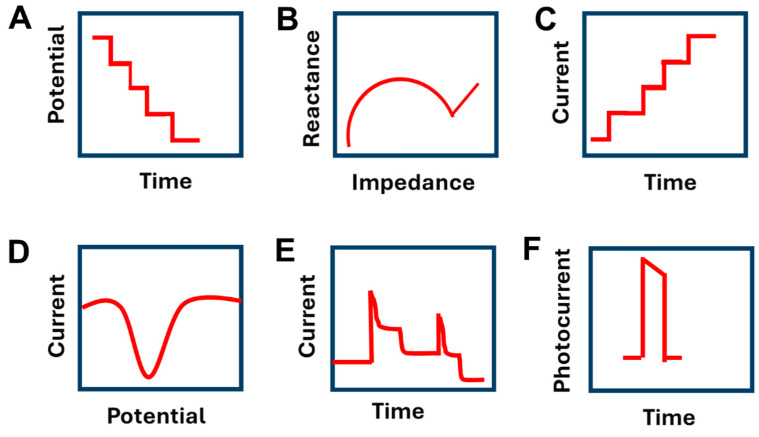
Schematic representation of (**A**) potentiometric, (**B**) impedimetric, (**C**) amperometric, (**D**) voltammetric, (**E**) organic electrochemical transistor, and (**F**) photoelectrochemical as electrochemical methods used for the detection of sweat biomarkers.

Sweat biomarkers can be detected using optical methods such as colorimetry [67,68,69], fluorescence spectroscopy [60,70], and SERS [71], as well as electrochemical methods [72]. The electrochemical ones include potentiometry [73], electrochemical impedance spectroscopy [74], amperometry [75,76], voltammetry [77], organic electrochemical transistor (OET) [78], and photoelectrochemical [62]. A potentiometric sensor is utilized for detecting the concentration of the targeted analyte via measurement of the potential when no current passes [79]. An impedimetric sensor can determine electroactive and electro-inactive substances via the measurement of electron transfer between the redox species and the electrode surface [80]. An amperometric sensor is appropriate for detecting single substances. In an amperometric sensor, the current is determined at a fixed potential. The current varies proportionally with the concentration of the analyte [81]. A voltammetric sensor is based on measuring current response within a specific range of potential values [14]. An OET, a three-terminal device, is composed of an organic semiconductor channel connecting source and drain electrodes, as well as a gate electrode linked to the channel through the electrolyte. The electrical signals from the gate electrode were amplified, leading to a sensitive determination of analytes [82]. Photoelectrochemical sensors merge the merits of optical and electrochemical sensors, where the absorption of light energy leads to an electrical signal [83]. The electrochemical responses of different electrochemical sensors are shown in Figure 1. Additionally, both advantages and disadvantages of different electrochemical sensors are discussed in Table 1. Amperometric and voltammetric sensors exhibit a high sensitivity and selectivity compared to other types of electrochemical sensors. However, they consume the analytes, which negatively affects the electroanalysis of low concentrations [84]. On the other hand, potentiometric sensors, which do not consume the target analytes, are used only for the detection of free ions [84,85,86]. Additionally, impedimetric sensors have some drawbacks, such as nonspecific interactions with the biomolecules affecting the response of the sensor [87]. Moreover, photoelectrochemical sensors, which have high sensitivity, exhibit poor stability for the bioanalysis [88,89,90].

Many electrode modifiers, such as conductive polymers [57,61], MIPs [91], graphene [92], graphene oxide (GO) [93], reduced graphene oxide (rGO) [94,95], dyes [96], and metal complexes [77], were tested as electrode modifiers for boosting the performance of sensors in the detection of sweat biomarkers (Table 2). Conductive polymer-modified electrodes show flexibility, high conductivity, and good mechanical features, while MIP-modified electrodes exhibit a high specificity and reproducibility [57,92]. Additionally, graphene-modified electrodes are known for their boosted electrical conductivity, increased surface area, and high electrocatalytic activity [93,94,95]. Moreover, conductive dyes and metal complexes were also examined as conductive materials for improving the determination of sweat biomarkers [77,96]. Moreover, nanostructures such as noble metals [97,98,99,100], metal oxides [101,102], MXene [103,104,105], nanofibers [106], CNTs [52], and multiwalled carbon nanotubes (MWCNTs) [107] showed excellent performance in the electrochemical detection of sweat biomarkers due to their increased surface area. In this review, a significant amount of focus is placed on MOF-based NCs as promising electrode modifiers for detecting sweat biomarkers with high performance.

## 4. Fabrication and Characterization of MOFs and Their Nanostructures

**Figure 2:**
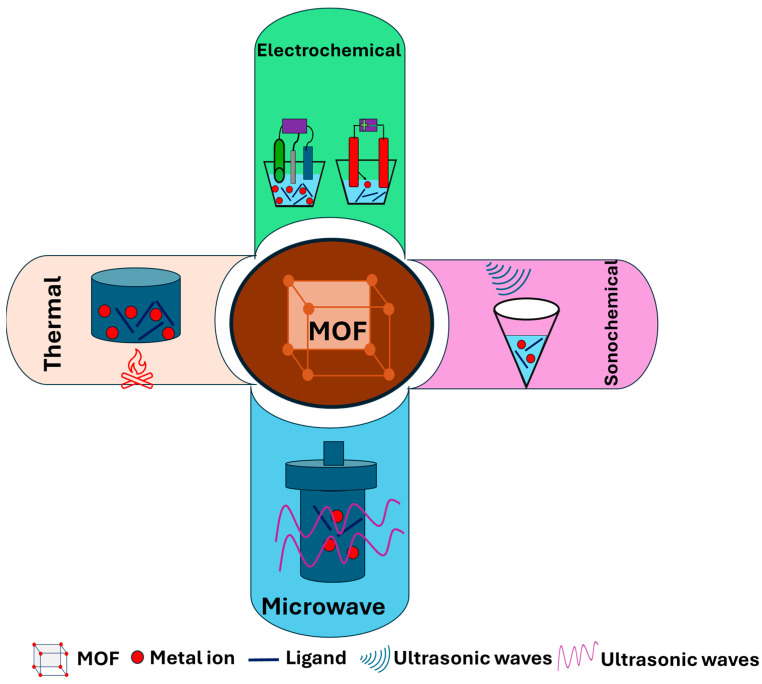
Schematic representation of different pathways used for the synthesis of MOFs.

MOFs with diverse crystal structures could be synthesized via many methods, such as hydrothermal/solvothermal, sonochemical-assisted synthesis, microwave-assisted synthesis (MAC), mechanochemical, and electrochemical synthesis [108]. In thermal methods, the metal salt and organic ligand are dissolved in water or organic solvent in the reaction vessel. Then, the reaction mixture is heated or autoclaved under elevated pressure for a specific time. Sonochemical-assisted synthesis is a green route for the preparation of MOF in which ultrasound is applied [109]. In MAC, the substrate and the appropriate solvent are mixed in a sealed Teflon vessel and then heated in the microwave for a suitable time. This method accelerates the nucleation during the synthesis of MOF structures, leading to a homogenous distribution of the particles [110]. In mechanochemical synthesis, an external force is utilized for the mechanical breakage of intramolecular bonds in MOF structures along with chemical modification [110]. The electrochemical methods can be used for the synthesis of MOF structures via anodic dissolution or cathodic deposition. The electrochemical synthesis is simple and quick, making it more convenient compared to the other methods [108]. Figure 2 shows a schematic representation of different methods used for the synthesis of MOF structures. Additionally, the MOF nanostructures, such as MOF NPs [111] and MOF nanosheets [112], can be obtained by controlling the synthesis conditions. Moreover, they can be prepared by mixing the bulk MOF with the NPs to be used as the electrode layer [64,113]. The formation and characterization of MOF nanostructures can be studied using various tools such as X-ray diffraction (XRD), Fourier transform infrared (FT-IR) spectroscopy, N_2_ adsorption–desorption isotherms, scanning electron microscopes (SEM), and transmission electron microscopes (TEM). XRD is necessary for examining the crystallinity degree of MOF nanostructures, while FT-IR is utilized for determining their chemical structures. N_2_ adsorption–desorption isotherms give valuable information about the surface area and porosity of the MOF nanostructures. Additionally, SEM and TEM are used for the determination of their size and morphology [114,115]. Moreover, their electrocatalytic properties are evaluated using cyclic voltammetry (CV), square wave voltammetry (SWV), and electrochemical impedance spectroscopy [116].

## 5. MOFs for Electrochemical Detection of Sweat Biomarkers

**Figure 3:**
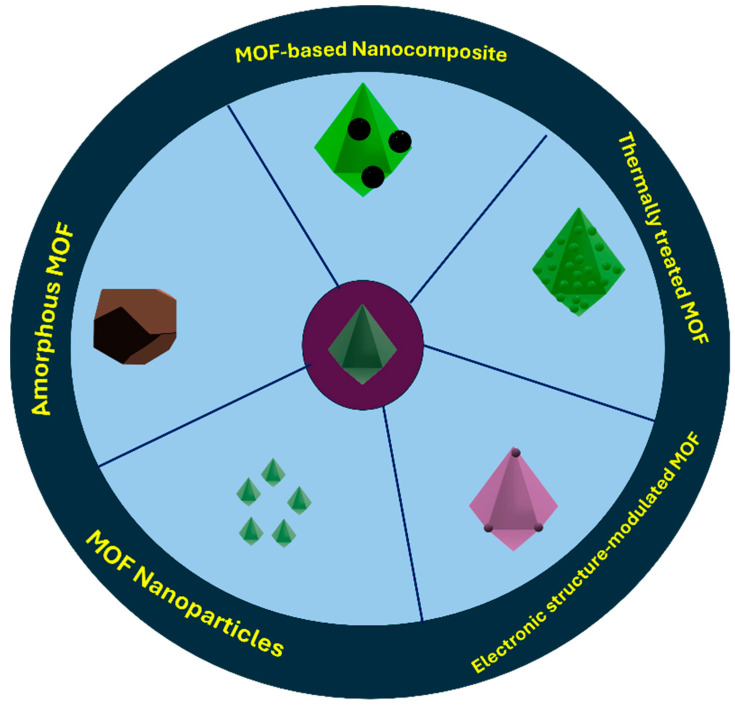
Schematic representation of different conductive MOF structures.

The high porosity and increased surface area of MOF structures assist in concentrating the analyte, leading to an increment in the strength of the electrochemical signal and enhancement of the sensor sensitivity as well as improved catalytic capacity [117]. Unfortunately, most MOFs have poor conductivity. Thus, the synthesis of MOF structures of high conductivity is necessary not only for the electrochemical sensors but also for their implementation in fuel cells, supercapacitors, and batteries [118]. The enhancement of the conductivity of MOFs could be achieved via many pathways such as the pyrolysis of MOFs, the synthesis of MOF structures with crystal defects (amorphous MOFs), enhancing metal-centered electronic structure, introduction of conductive materials into MOF structures, or the synthesis of MOF NPs (Figure 3). Pyrolysis of MOFs is based on sustaining the required porous structure, facilitating the molecular transport, and making the active sites highly dispersed [119]. Amorphous MOFs can be obtained via thermal treatment, ball milling, or high-pressure treatment of the crystalline MOFs [120,121]. They could be directly synthesized by the selection of suitable metal and ligand precursors as well as variation of the synthesis conditions [122]. Additionally, the metal centers in MOF can influence the layer spacing of π-conjugation, enhancing the electron movement and providing more active sites [123].

**Figure 4:**
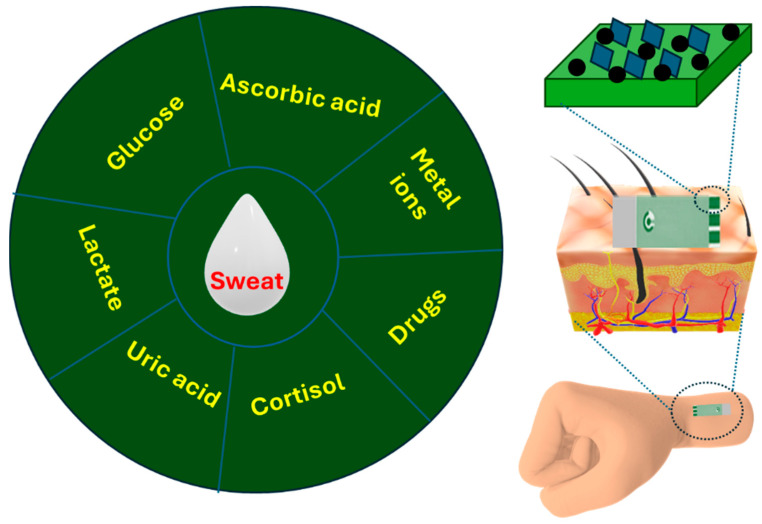
Schematic representation of sweat biomarkers detected by MOF-based NCs.

Shortly, amorphous or low-dimensional MOFs could be used to prepare monolayer-modified electrodes for the effective electrochemical determination of sweat biomarkers (Figure 4) due to their high electrocatalytic conductivity. On the other hand, crystalline MOF monolayer-modified electrodes have poor conductivity. Thus, other conductive materials such as NPs or polymers should be immobilized onto the MOF layer-modified electrodes. The high electrical conductivity of the electrodes modified with MOF NCs is necessary for fabricating wearable and flexible sweat sensors of high sensitivity. For example, CNTs were previously used as an electroactive coating of MOF NC-modified electrodes, which endowed the wearable sensors with high performance for glucose and cortisol detection with a lower detection limit [124,125]. Additionally, the ultrathin nanosheets in NiMn-MOF improved the electronic structure, which enhanced the electrical conductivity of the MOF NC-based wearable sensor [126]. Moreover, the electrical conductivity of CuNi-MOF NC was enhanced after doping with reduced rGO for its application in wearable sweat uric acid and dopamine sensing [127].

Table 3 explains the electrochemical responses of different MOF NCs used for the electrochemical sensing of sweat biomarkers. In MOF NCs, MOFs can be used as an electrocatalyst, an adsorbent of the target sweat biomarker, or a matrix for the electroactive NPs [41,112,128,129,130,131]. The electrode material, the electrochemical technique, and the limit of detection of the MOF NC-based electrochemical sensors used for the determination of sweat biomarkers are listed in Table 4.

## 6. MOF-Based NCs for Electrochemical Detection of Sweat Biomarkers

### 6.1. Cu-MOF

Cu-MOFs can be used in many applications, such as pollutant removal [132,133], hydrogen production [134], optical sensors [135,136], and electrochemical sensors [29,45,137]. The role of Cu-MOF NCs in the detection of sweat biomarkers was previously reported.

#### 6.1.1. Cu-MOF Nanostructures

Cu-MOF, a nanorime-structured Cu(INA)2, was incorporated with activated carbon fiber and rGO for detecting glucose and lactate in sweat using amperometry. The porous rime-structured MOF enabled the entrapment of analytes and improved the electron movement inside the pores [130]. Notably, Cu-MOF, nanorime-structured Cu(INA)2, showed the lowest detection limits for determining lactate and glucose in sweat among other MOF-based NCs [56,124,128,129,130]. This is attributable to the synergistic effects of nanorime MOF, activated carbon fiber, and reduced graphene oxide.

#### 6.1.2. Cu-MOF/Pt NPs

Cu-MOF was incorporated with platinum (Pt) NPs and lactate oxidase at a screen-printed electrode for the preparation of an enzymatic sensor to electrochemically detect lactate in sweat. The Pt NPs were used due to their catalytic effect, while Cu-MOF was used for enhancing signal sensitivity and stability [129].

#### 6.1.3. Cu-CAT) Nanowires

Moreover, Cu-catecholate (Cu-CAT) nanowires were utilized for the increment in the active surface area and enhancing the electron transfer rate for improving the detection of glucose and lactate in sweat on the basis of their oxidation using amperometry [56].

#### 6.1.4. Cu-MOF Nanosheets

Cu-MOF nanosheets have been used as a glassy carbon electrode (GCE) modifier for determining ascorbic acid in sweat based on its oxidation, where two electrons and two protons were transferred. The Cu-MOF nanosheets with unsaturated active sites enabled ascorbic acid adsorption, and their increased surface area was essential for the electrocatalytic oxidation of ascorbic acid [75]. Among MOF-based NCs, Cu-MOF nanosheets showed the lowest detection limits for ascorbic acid determination in sweat [62,75,111].

### 6.2. Ni-MOF Nanorods

**Figure 5:**
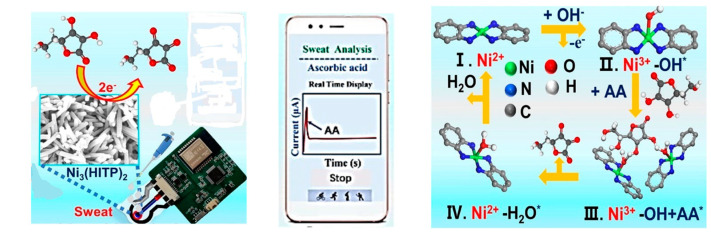
Schematic illustration of electrocatalytic oxidation mechanism of ascorbic acid by Ni‒MOF‒based electrochemical sensor (reprinted from ref. [111] with permission from El‒Sevier).

Ni-MOF structures were studied for catalyzing oxygen evolution reactions [138] as well as enhancing the performance of supercapacitors [139]. Additionally, they are considered to be a promising electrocatalyst for use in electrochemical sensors [20]. For example, the highly crystalline and conductive Ni-MOF, (Ni_3_(2,3,6,7,10,11-hexaiminotriphenylene)_2_, Ni_3_(HITP)_2_) nanorods, have been utilized as electrocatalysts for the electrochemical determination of ascorbic acid in sweat via its oxidation using amperometry technique. The electrochemical sensor has been integrated with a smartphone for monitoring the ascorbic acid signal and used as a wearable sensor [111], as shown in Figure 5. Additionally, 2D Ni-MOF nanosheets have been used as electrocatalysts and have been incorporated with activated flexible carbon cloth and polypyrrole NPs for detecting Ni (II) ions in sweat using potentiometry. The Ni (II) ions were adsorbed into the porous structure of MOF, enhancing the selectivity of the potentiometric sensor [112].

### 6.3. NiCo-MOF/CNTs

**Figure 6:**
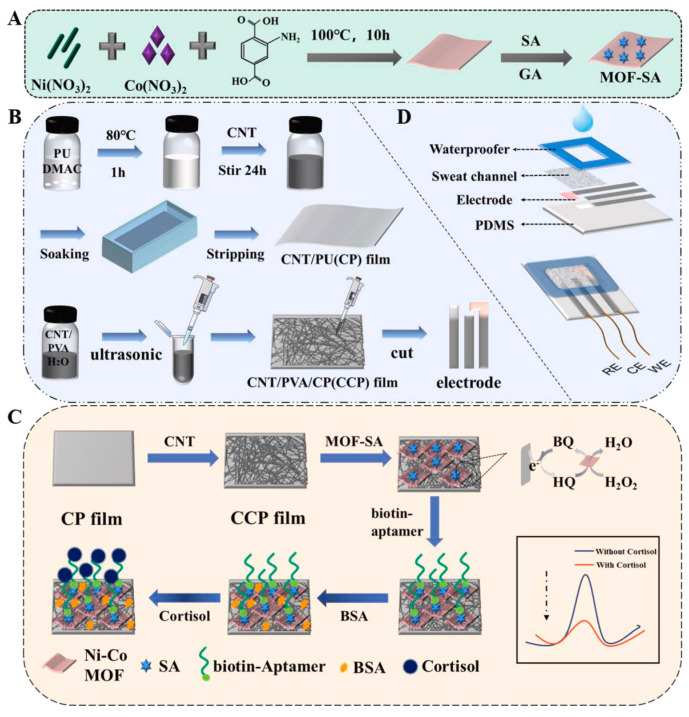
Schematic illustration of the (**A**) synthesis of NiCo‒MOF, (**B**) preparation of NiCo‒MOF NC‒modified electrode, (**C**) electrochemical determination of cortisol detection, and (**D**) composition of cortisol sensor patch (reprinted from ref. [125] with permission from El-Sevier).

NiCo-MOFs have been used in the construction of supercapacitors [28,140] and hydrogen production [141], as well as electrochemical sensors [142]. Recently, NiCo-MOF has been introduced into electrochemical sensors to detect cortisol and glucose in sweat [124,125]. NiCo-MOF nanosheets have been functionalized with CNTs and polyurethane for detecting cortisol in sweat. The detection process relied on the high catalytic activity of NiCo-MOF towards hydroquinone oxidation, which led to an enhancement in the redox current signal. The MOF was attached to a cortisol aptamer for specific binding with cortisol. The redox current signal was monitored using differential pulse voltammetry (DPV) [125]. Figure 6 shows an illustration of the synthesis of NiCo-MOF and its composite used for the detection of cortisol. Additionally, the enzyme-like NiCo-MOF has been used as an electrocatalyst for detecting glucose in sweat using the amperometry technique. The conductivity of the sensor was enhanced by incorporating MWCNTs and CNTs into the electrode surface. The Ni(III)/Ni(II) and Co(IV)/Co(III) conversions irreversibly catalyzed the oxidation of glucose to gluconolactone [124].

### 6.4. Zeolitic Imidazolate Framework (ZIF)

The zeolitic imidazolate framework (ZIF), an MOF with zeolite-like structure, is a highly ordered porous material with exceptional features that make it desirable for many applications [143,144]. For example, it has been used as an adsorbent to eliminate environmental pollutants [16,145] as well as a catalyst for the cycloaddition of CO_2_ [146]. ZIF-8, which is composed of tetrahedral coordination of zinc atoms linked by 2-methylimidazole, and ZIF-67, 2-methylimidazole cobalt salt have been used in the determination of sweat biomarkers. ZIF-8 plays a significant role in improving enzyme immobilization in enzymatic electrochemical sensors [64].

#### 6.4.1. ZIF-8/Au NPs

ZIF-8 was used for the encapsulation of gold (Au) NPs and glucose oxidase into their cavities for the catalytic oxidation of glucose in sweat using amperometry. The sensitivity of the electrochemical sensor showed a 10-fold increase after the incorporation of Au NPs into the system [131].

#### 6.4.2. ZIF-8/Ag NPs

ZIF-67 mixed with silver (Ag) NPs has been used for the electrochemical detection of chloride ion (Cl^−^) in sweat, where ZIF-67 provides protection for Ag NPs. The determination of Cl^−^ was based on the reduction in the Ag NP oxidation current, which was monitored by DPV in the presence of Cl^−^ [41].

#### 6.4.3. ZIF-8 NPs/GO

**Figure 7:**
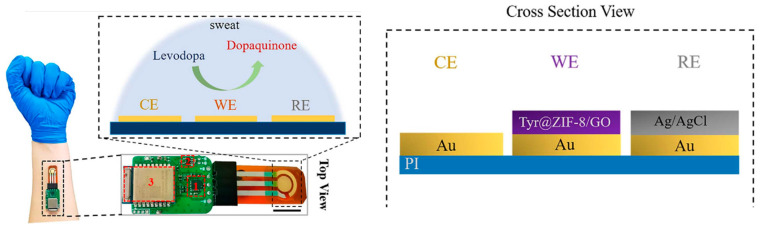
A schematic representation of a wearable levodopa sweat sensor, where 1, 2, and 3 refer to microcontroller, analog front-end, and Bluetooth transceiver (reprinted from Ref. [64] with permission from El‒Sevier).

Additionally, ZIF-8 NPs mixed with GO have been used for efficient immobilization of tyrosinase enzyme for detecting the levodopa drug in sweat using amperometry technique (Figure 7). The electrochemical sensor has been integrated with a wireless communication device, which enables its portability [64].

#### 6.4.4. ZIF-67-Derived NiCo

ZIF-67-derived NiCo layered double hydroxide (LDH) nanocage was used as an electrocatalyst for the non-enzymatic detection of lactate in sweat via its amperometric oxidation. Co and Ni with multiple valencies act as redox mediators in the MOF structure [128].

### 6.5. Zn-TCPP Nanosheets/MWCNTs

**Figure 8:**
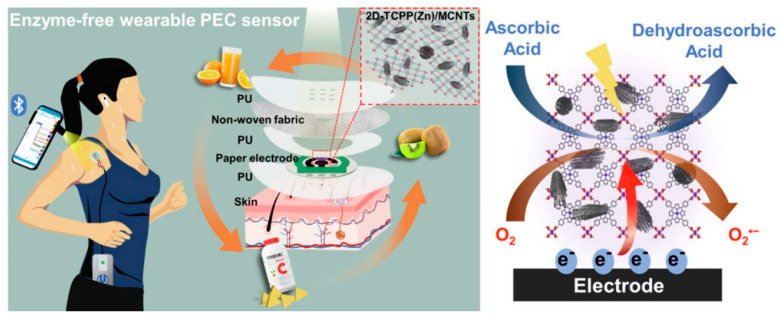
A schematic illustration of a wearable electrochemical sensor for sweat ascorbic acid determination (reprinted from Ref. [62] with permission from El‒Sevier).

Zn-MOF was used for the determination and removal of pollutants from water [147]. It can also be used in the electrochemical detection of sweat biomarkers. Recently, the two-dimensional Zn porphyrinic MOF, Zn-5,10,15,20-tetrakis (4-carboxyphenyl) porphyrin (Zn-TCPP), nanosheets have been incorporated with MWCNT for detecting ascorbic acid in sweat using photoelectrochemical technique. Zn-TCPP generates electron–hole pairs under the stimulation of a light source, and MWCNT enhances the electron transfer efficiency of Zn-TCPP [62]. Figure 8 exhibits the PEC sensor and the mechanism of ascorbic acid determination.

## 7. MOF NCs as Promising Electrode Modifiers for Detecting Sweat Biomarkers

### 7.1. Two-Dimensional Co-MOF Nanostructures

Co-MOFs have been utilized in wastewater treatment [148], the fabrication of supercapacitors [149], and catalysis [150,151]. Recently, they have been studied as electrocatalysts for uric acid detection. The two-dimensional Co-MOF NC, which was adsorbed on carbon cloth, provided a large specific area and highly exposed active sites, which enhanced the electrocatalytic activity toward uric acid oxidation in biological samples. Additionally, it showed the lowest detection limit among the different MOF structures used for determining uric acid in biological samples [152].

### 7.2. Cu-MOF

#### 7.2.1. Cu-MOF Nanostructures

Cu-MOFs were examined for determining uric acid and glucose in biological samples. For example, Cu-benzene-1,3,5-tricarboxylic acid (Cu-BTC), as a good electrocatalyst, was used as a modifier of carbon paste electrode to detect uric acid based on its electro-oxidation via DPV technique [153]. Additionally, the porphyrinic MOF, Cu-TCPP, showed high electrocatalytic activity toward the oxidation/reduction of uric acid. Therefore, Cu-TCPP MOF-modified GCE was used for the detection of uric acid using CV, DPV, amperometry, and photoelectrochemical techniques [154].

#### 7.2.2. Cu-MOF/Ni NPs

Cu-MOF was decorated with Ni NPs for sensitive detection of glucose using the amperometry technique. The synergistic effect of Ni NPs and Cu-MOF-C enhanced the electrocatalytic activity of the sensor for detecting glucose compared to single Cu-MOF-C or Ni NPs [55].

#### 7.2.3. Cu-MOF/Pt NPs

Cu-MOF was previously decorated with Pt NPs for detecting glucose in biological samples. The high electrocatalytic activity of Ni NPs boosted the performance of the electrochemical sensors [155]. The Cu-MOF incorporated with Ni NPs showed the lowest detection limits among other promising MOF-based NCs that were used for detecting glucose in biological samples [55,156].

#### 7.2.4. CuCo Nanostructures

The bimetallic CuCo MOF nanostructures were used as electrocatalysts for the sensitive detection of glucose in biological samples due to an enhancement in electrochemical sensing after bimetallic synergy [156].

#### 7.2.5. CuCo-MOF/Cu NPs

The incorporation of CuCo-MOF into Cu NPs was examined to detect glucose in biological samples using amperometry. The presence of Cu NPs enhanced the electrical conductivity of the MOF structure, which boosted the kinetics of glucose determination [157].

#### 7.2.6. CuCo-MOF/CuO NPs

Cu-MOF can be co-precipitated with Co-BTC to form a CuCo MOF electrocatalyst in the presence of CuO nanowires, which act as a framework for the growth of MOF for the determination of glucose [158].

### 7.3. MnCO-MOF Nanostructures

MnCo-MOFs were utilized in fields such as adsorption [24], supercapacitors [159], and catalysis [160,161]. Additionally, they can be utilized in the electrochemical determination of glucose in biological samples. MnCO-MOF-74 was used for the preparation of MnCO-based NC with hierarchical carbon via carbonization for the determination of glucose based on its oxidation using amperometry. MnCO-based NC showed favorable electrocatalytic activity due to its increased specific surface area with multiple active sites [162].

### 7.4. Nb-MOF Nanostructures

Nb-MOFs were utilized in many fields such as supercapacitors [163] and optical sensors [164]. Recently, Nb-BTC supported with carbon nanofibers has been used as an electrocatalytic active NC for detecting uric acid in biological samples using the DPV technique. Nb is an electroactive, conductive, stable, and biocompatible element. Thus, it was beneficial for improving the performance of uric acid detection [165].

### 7.5. Ni-MOF

#### 7.5.1. Ni-MOF Nanostructures

Ni-MOF NCs were extensively developed for detecting glucose, lactate, and uric acid in biological samples. For example, Ni-MOF nano-flowers with multi-nanoneedle-like structures exhibited excellent electrocatalytic activity towards lactate electrooxidation using the amperometry. They have an increased surface area, facilitating the adsorption of lactate and electron movement between immobilized lactate and the working electrode [166]. Similarly, Ni-MOF nanosheets were used for detecting glucose in biological samples using the amperometry technique. The nanosheets provided more active sites, which improved the catalytic activity of the electrochemical sensor [167]. The nanoflocculent Ni-MOF, which was prepared by combining a carbonized loofah sponge with Ni-MOF, has been used for the electrochemical detection of glucose due to its improved conductivity [168].

#### 7.5.2. Ni-MOF NPs

Glucose can also be detected using a Ni NPs/carbon/graphene composite, which resulted from the pyrolysis of Ni-BTC via its amperometric oxidation. The presence of graphene enhanced the dispersion of Ni NPs and the conductivity of the NC [169].

#### 7.5.3. Ni-MOF/Au NPs

Ni-MOF as an electrocatalyst was decorated with Au NPs to improve the determination of uric acid in biological samples based on its oxidation using the DPV technique. Ni-MOF provided Ni^2+^/Ni^3+^ redox couple, while the presence of Au NPs increased the electrical conductivity of the NC [170].

#### 7.5.4. Ni-MOF/CNTs

Ni-MOF was functionalized with conductive CNTs to increase the electrocatalytic activity of Ni-MOF for the detection of glucose [20].

#### 7.5.5. Ni_2_P/C NPs

Ni-MOF-74 can be calcinated with red phosphorus to form Ni_2_P/C NC with a good electron transfer efficiency to be used as a GCE modifier for detecting uric acid. The efficacy of Ni_2_P/C NC may be attributable to the synergistic effect between the good electrical conductivity of porous carbon and the catalytic effect of Ni_2_P NPs [171].

#### 7.5.6. Ni-P NPs

The phosphidation of Ni-MOF was carried out to synthesize phosphide NPs, Ni-P, as a new electrocatalyst for glucose sensing due to their increased surface area and improved conductivity [38].

### 7.6. NiCo-MOF/Au NPs

NiCo-MOF nanocubes were previously incorporated with tin sulfide (SnS_2_) nanosheets and Au NPs into an electrochemical immunosensor for detecting cortisol. They increased the spacing between the layered structures of SnS_2_ nanosheets, leading to an effective reduction in the aggregation of SnS_2_. Additionally, they provided more active sites to deposit Au NPs. The formed NC enhanced the sensor signal, which was monitored by SWV technique. In the presence of cortisol, the peak current was reduced because of the electrochemical inactivity of cortisol that hampers the movement of electrons [50].

### 7.7. NiMn-MOF Nanostructures

NiMn-MOF NCs can be utilized in supercapacitors [172,173] and batteries [174]. Additionally, they can be used in the electrochemical assessment of biological substances. For example, a NiMn-layered double hydroxide (LDH)-based MOF with multiple active sites was used for sensing glucose based on its electrooxidation using the amperometry technique. The excellent electrocatalytic activity is attributable to the synergistic effect between Ni and Mn [175].

### 7.8. ZIF

ZIF-based NCs were developed for determining uric acid, lactic acid, and glucose in different biological samples.

#### 7.8.1. ZIF Nanoporous Carbon

Both ZIF-8 and ZIF-67 can be transformed into porous carbon, providing more catalytic active sites and enhanced electron movement for detecting uric acid using DPV technique [176]. Similarly, bimetallic ZIFs containing Zn/Co NPs were transformed into nanoporous carbon to be used as an electrocatalyst for assessing uric acid and ascorbic acid using the DPV technique [177].

#### 7.8.2. ZIF/Pt NPs

ZIF-8 was used to distribute Pt NPs within its porous structure to form an electroactive NC for determining uric acid. The uric acid was detected based on its oxidation using DPV technique [178].

#### 7.8.3. ZIF/ZnO NPs

ZIF-67 can be used for preparing Co-doped N-containing carbon framework (Co-NCF) NC for detecting lactic acid using amperometry. The catalytic activity was attributed to the presence of Co NP. Additionally, a ZnO nanomembrane and carbon NPs were used for induction of MOF film growth and formation of the conductive pathway within the carbon skeleton, respectively [179].

#### 7.8.4. ZIF-67 Derived NiCo LDH Nanosheets

ZIF-67-derived NiCo LDH nanosheets as electroactive materials were combined with cobalt carbonate nanorods as a template for enhancing the electrochemical performance of non-enzymatic glucose sensors. The electrocatalytic activity of ZIF-67-derived NiCo LDH was attributed to the presence of Ni^2+^/Ni^3+^ and Co^2+^/Co^3+^ redox mediators [180].

### 7.9. Zn-MOF Nanoflower

A Zn-MOF nanoflower, which was incorporated with Ce ions, was utilized for determining uric acid in biological samples based on its oxidation. The presence of Ce ions enhanced the MOF charge conduction, which improved the sensor’s sensitivity [181].

### 7.10. Zr-MOF (Porous Carbon-ZrO_2_ NPs)

Zr-MOFs have been used for enhancing photocatalytic degradation [182] as well as optical and electrochemical sensing [183,184]. Previously, Zr-MOF was calcinated to ZrO_2_ porous carbon frameworks to be used as a GCE modifier for detecting uric acid in biological samples. The electrocatalytic activity of the electrochemical sensor was attributed to ZrO_2_ NPs distributed in the carbonaceous network [22]. All the promising MOF-based NCs, which can be used for the detection of sweat biomarkers, are listed in Table 5.

## 8. Perspective, Challenges, and Future Limitations

The use of the electrochemical detection of sweat biomarkers for the early diagnosis of disease is developing quickly. Currently, there are many wearable electrochemical sensors for real-time monitoring of the biomarkers and rapid display of the results, despite space and time restrictions. Various electrode modifiers could be incorporated into the electrochemical sensors to enhance their performance. Among these modifiers, MOF nanostructures are effective materials for the electrochemical determination of sweat biomarkers. However, there are some challenges such as long-term stability, sensitivity, selectivity, and biocompatibility that should be addressed when using MOF materials. The poor stability of some MOF NCs, which affect the sensitivity of the sensor, results from the weak interaction between the MOF NC-based modifier and the electrode surface. Additionally, the poorly stable MOF NC-modified electrodes negatively affect the reproducibility of the electrochemical responses. Therefore, a binder should be added to improve MOF NC/electrode interaction. Unfortunately, binders may negatively affect the conductivity of the electrodes. So, a lower concentration of binder should be added. The stability and reproducibility of the modified electrodes could be enhanced by introducing MOF materials into a highly stable matrix. Additionally, the selectivity limitation can arise from the non-specific adsorption of non-targeted substances due to the large specific surface area. Thus, fine-tuning the pore structures of MOFs is essential for the selective detection of the target substances. Moreover, the biocompatibility of MOF NCs and the absence of toxic materials are crucial for their applications in wearable sensing. Therefore, many studies have reported the incorporation of biomacromolecules such as chitosan and cellulose and synthetic materials such as GO and nanofibers into MOF NCs for achieving the biocompatibility of wearable sensors.

MOF-based NCs facilitate the development of wearable sweat sensors, as they have a high sensitivity and selectivity. However, the most investigated MOF-based NCs were introduced into enzyme-free electrochemical sensors, which still have some challenges, such as selectivity when they are used for detecting substances with similar structures. On the other hand, enzyme-free electrochemical sensors have shown high sensitivity compared to enzymatic or immune sensors. Additionally, the detection of some sweat biomarkers, such as glucose, requires basic conditions that cause epidermal monitoring troublesome. Therefore, researchers are still working on optimizing the performance, sensitivity, appropriate stability, and selectivity of these electrochemical sensors. The incorporation of MOF-based NCs into flexible sweat wearable sensors recently gained much attention. These sensors are considered smart health monitoring tools that collect information easily.

## 9. Conclusions

MOF-based NCs are considered appropriate electrode modifiers for improving the electrochemical detection of sweat biomarkers. They could be applied in electrochemical sensing as they have brilliant electrical conductivity compared to the pristine MOFs. They are obtained directly by the synthesis of MOF in the nanoform or by combining the MOF with nanostructures such as metal NPs, metal oxide NPs, MWCNTs, and nano-polymers. These NCs are directly immobilized onto the electrode surface through ex situ methods such as drop casting. Alternatively, the MOF-based NCs can be formed on the surface of the electrode through in situ methods such as anodic dissolution or cathodic deposition. The electrodes modified with MOF-based NCs were examined for sensing many sweat biomarkers such as metal ions, glucose, ascorbic acid, uric acid, lactate, and cortisol. The MOF structures, which are known for their high porosity and increased surface area, concentrate the target analyte to enhance the electrochemical signal. In this review, we list the previously tested MOF-based NCs for the detection of sweat biomarkers. Additionally, the promising MOF-based NCs that can be applied to detect sweat biomarkers are highlighted. Overall, MOF-based NCs can be incorporated into flexible sweat sensors for real-time monitoring of different biomarkers.

## Figures and Tables

**Table 1:** Advantages and disadvantages of electrochemical sensors.

Type of Electrochemical Sensor	Advantages	Disadvantages	Comments	Ref
Amperometric	The fixed potential during amperometric detection results in a negligible charging current.	It leads to the consumption of the analyte that may result in hindering the measurements at low concentrations.		[76,84]
Impedimetric	It is a simple, sensitive, and nondestructive method. The sensor can be miniaturized into devices.	The nonspecific interaction of biomolecules affects the sensor response.		[87]
Organic electrochemical transistor	It is easy to be designed and does not need a high voltage for operation.	It has high hardness and a large mass.	The fiber-based organic electrochemical transistors have been used to overcome the limitation of large mass.	[78]
Potentiometric	It has a fast response and an ability for miniaturization and integration. There is no consumption of the target analyte during the electroanalysis.	The signal of the potentiometric sensor depends on temperature and determines the free ions only.		[73,84,85,86]
Photoelectrochemical	It is known for its simple instrumentation, low cost, and miniaturization, low background, and high sensitivity.	It has poor stability for bioanalysis.	This stability limitation can be overcome by using an enzyme-free system	[88,89,90]
Voltametric	It shows superior sensitivity, robustness, and selectivity	It leads to the consumption of the analyte that may result in hindering the measurements at low concentrations		[5,84]

**Table 2:** Types of electrocatalysts used for sensing sweat biomarkers.

Electrocatalyst	Example	Sweat Biomarker	Catalytic Mechanism	Comments	Ref
Conductive polymers	PEDOT	Lactate	Lactate was oxidized by lactate oxidase to form pyruvate and hydrogen peroxide. Then, hydrogen peroxide was decomposed into hydrogen ions, which were oxidized at the PEDOT interface producing a current response.	Conductive polymers are flexible and conductive with acceptable mechanical properties.	[57]
MIPs		Lactate		They are specific and inexpensive with high reproducibility.	[92]
Graphene-based materials	Graphene	K+		The electrocatalytic activity of graphene is attributed to its superior conductivity and large surface area.	[93]
	Graphene oxide	Glucose -Lactate		The high number of oxidized moieties are considered active sites for the interaction with the target analytes.	[94]
	Reduced -graphene oxide	pH—K+			[95]
Dyes	Prussian Blue	H_2_O_2_	The reduced form of Prussian blue, Prussian white, catalyzed the reduction of hydrogen peroxide at low potential.		[96]
Metal complexes	Cu(II)-complex	Glucose	1-Reduction of Cu(II) in the complex to metallic Cu by applying a negative potential 2-Oxidation of glucose by the electrogenerated Cu(II) during DPV scan		[77]

MIPs: molecularly imprinted polymers, PEDOT: poly(3,4-ethylenedioxythiophene).

**Table 3:** Electrochemical behavior of different MOF NCs for the detection of sweat biomarkers.

MOF	NP/NC	Electrochemical Behavior of MOF NC	Comments	Ref
Cu-MOF	Pt	Cu-MOF enhanced the signal’s sensitivity and stability, while Pt allowed higher sensitivity for detecting sweat biomarker due to its high electrocatalytic activity.	The chitosan layer was added as a biocompatible cationic polymer for enzyme immobilization.	[129,130]
	Cu-MOF	Cu-MOF acted as an electrocatalyst by increasing the electrochemically active surface area and enhancing the electron transfer rate.		[56,75,130]
Ni-MOF	Ni_3_(HITP)_2_	Ni-MOF NPs showed high electrocatalytic activity resulting from the effects of highly active Ni-N4 catalytic sites, the two-dimensional superimposed honeycomb lattice of Ni-MOF, and the increased surface area.		[111]
	Porous carbon, polypyrrole, Ni-MOF	Ni-MOF NC acted as an electroactive material and adsorbent for the sweat biomarker.	Ni-MOF was incorporated with porous carbon cloth decorated with nitrogen.	[112]
NiCO-MOF	NiCo-MOF, CNTs	NiCo-MOF efficiently captured the aptamer of sweat cortisol owing to its high specific surface area.		[125]
	CNTs, MWCNT	NiCo-MOF exhibited high electrocatalytic activity and optimized sensitivity for sweat biomarkers.	NiCo-MOF NC showed high stability under stretching and bending conditions.	[124]
ZIF-8	Au NPs	The thermally and chemically stable ZIF-8 was used to encapsulate the enzyme and NPs. The presence of conductive Au NPs boosted the activity of the enzyme and improved electron transfer.		[131]
ZIF-67	ZIF-67 derived NiCo LDH	ZIF-67 acted as an electrocatalyst for enhancing the oxidation of sweat biomarker.		[128]
	ZIF-67, Ag NPs	ZIF-67 provided good dispersity as well as a protective effect for Ag NPs.		[41]
Zn-MOF	Zn-TCPP- MOF, MWCNT	MOF NC generated electron-hole pairs under the stimulation of a light source.		[62]

**Table 4:** MOF-based NCs for electrochemical detection of sweat biomarkers.

MOF	NPs	Electrode Material	Sweat Biomarker	Electrochemical Technique	LOD	Ref
Cu-MOF	Pt	Lactate oxidase/Cu-MOF/chitosan/Pt NPs/SPE	Lactate	Amperometry	0.75 μM	[129]
	Cu-MOF	ACF-rGO/Cu(INA)_2_	Lactate	Amperometry	500 nM	[130]
			Glucose		50 nM	
	Cu-MOF	Cu-CAT nanowires/CP	Lactate	Amperometry	10 μM	[56]
			Glucose		2 μM	
	Cu-MOF	Cu-BDC/GCE	Ascorbic acid	Amperometry	0.1 μM	[75]
Ni-MOF (Ni_3_(HITP)_2_)	Ni_3_(HITP)_2_	Ni_3_(HITP)_2_ nanorods/SPCE	Ascorbic acid	Amperometry	1 μM	[111]
Ni-MOF	Porous carbon, polypyrrole, Ni-MOF	AFCC-polypyrrole NPs/2D Ni-MOF	Ni (II) ions	Potentiometry	2.7 × 10^−6^ M	[112]
NiCO-MOF	NiCo-MOF, CNTs	Cortisol/apt/NiCo-MOF/CNTs/PVA/CP	Cortisol	DPV	0.032 ng/mL	[125]
	CNTs, MWCNT	NiCo-MOF/CNTs/MWCNT/PDMS	Glucose	Amperometry	6.78 μM	[124]
ZIF-8	Au NPs	Glucose oxidase/Au NPs/ZIF-8/GCE	Glucose	Amperometry	50 nM	[131]
	ZIF-8	Tyrosinase/ZIF-8 NPs/GO/Au SPE	Levodopa drug	Amperometry	0.45 μM	[64]
ZIF-67	ZIF-67 derived NiCo LDH	ZIF-67 derived NiCo LDH nanocage/SPCE	Lactate	Amperometry	0.399 mM	[128]
	ZIF-67, Ag NPs	ZIF-67/Ag NPs/PDA/GCE	Cl-	DPV	1 mM	[41]
Zn-TCPP-MOF	Zn-TCPP- MOF, MWCNT	Zn-TCPP- MOF nanosheets/MWCNT/SPPE	Ascorbic acid	PEC	3.61 μM	[62]

ACF: Activated carbon fiber; AFCC: activated flexible carbon cloth; CNT: carbon nanotube; CuBDC: copper 1;4-benzenedicarboxylate; DPV: differential pulse voltammetry; GCE: glassy carbon electrode; GO: graphene oxide; MOF: metal–organic framework; MWCNT: multi-walled carbon nanotubes; Ni3(HITP)2: Ni_3_(2;3;6;7;10;11-hexaiminotriphenylene)_2_; NP: nanoparticle; PDA: polydopamine; PDMS: polydimethylsiloxane; PEC: photoelectrochemical; PVA: polyvinyl alcohol; rGo: reduced graphene oxide; SPCE: screen printed carbon electrode; SPE: screen printed electrode; SPPE: screen printed paper electrode; TCCP: 5;10;15;20-tetrakis (4-carboxyphenyl) porphyrin; ZIF: zeolitic imidazolate framework.

**Table 5:** MOF-based NCs as promising materials for the electrochemical detection of sweat biomarkers.

MOF	NPs	Electrode Material	Analyte	Role of MOF	Electrochemical Technique	LOD	Ref
Co-MOF	Co-MOF	Co-MOF/CC	Uric acid	Electrocatalyst	DPV	7 nM	[152]
Cu-MOF	Cu-BTC	Cu-BTC MOF/CPE	Uric acid	Electrocatalyst	DPV	0.2 μM	[153]
Cu-MOF	Cu-TCPP	Cu-TCPP MOF/GCE	Uric acid	Electrocatalyst	CV	0.03 μM	[154]
					DPV	1.37 μM	
					Amperometry	0.3 μM	
					Photoelectrochemical	0.01 μM	
Cu-MOF	Ni	Ni NPs/Cu-MOF-C/GCE	Glucose	Electrocatalyst	Amperometry	0.090 μM	[55]
Cu-MOF	Pt	Pt NPs/Cu-MOF/Au electrode	Glucose	Electrocatalyst	DPV	0.06 mM	[155]
CuCO-MOF	CuCO-MOF	CuCO MOF/CC	Glucose	Electrocatalyst	Amperometry	0.27 μM	[156]
CuCo-MOF	CuO	CuCo-BTC derivative/CC	Glucose	Electrocatalyst	Amperometry	0.09 μM	[158]
CuCo-MOF	Cu	Cu/CuCo MOF	Glucose	Electrocatalyst	Amperometry	0.27 μM	[157]
MnCo-MOF-74	MnCo	Co/MnO/hierarchical carbon/GCE	Glucose	Electrocatalyst	Amperometry	1.31 μM	[162]
Nb-MOF	Nb(BTC) MOF, CNF	Nb(BTC) MOF/CNF/GCE	Uric acid	Electrocatalyst	DPV	70 nM	[165]
Ni-MOF	Ni-MOF	Ni-MOF/Pt electrode	Lactate	Electrocatalyst	Amperometry	5 μM	[166]
Ni-MOF	Ni-MOF	Ni-MOF nanosheet/GCE	Glucose	Electrocatalyst	Amperometry	0.6 μM	[167]
Ni-MOF	Ni-MOF	CLS/Ni-MOF/GCE	Glucose	Electrocatalyst	Amperometry	0.4 μM	[168]
Ni-MOF	Ni	Ni/C/graphene	Glucose	Electrocatalyst	Amperometry	0.6 μM	[169]
Ni-MOF	Au	Au/Ni-MOF/GCE	Uric acid	Electrocatalyst	DPV	5.6 μM	[170]
Ni-MOF	CNTs	CNTs/Ni-MOF/GCE	Glucose	Electrocatalyst	Amperometry	0.82 μM	[20]
Ni-MOF-74	Ni_2_P/C	Ni2P/C/GCE	Uric acid	Electrocatalyst	DPV	70 nM	[171]
Ni-MOF	Ni-P	Ni-P/Ni foam	Glucose	Electrocatalyst	Amperometry	0.15 μM	[38]
Ni-CO MOF	SnS_2_, Ni-CO MOF, Au	BSA/anti-cortisol (C-Mab)/Au NPs/SnS2/Ni-CO MOF	Cortisol	Providing active sites for the deposition of Au NPs	SWV	29 fg/mL	[50]
Ni-Mn MOF	Ni-Mn MOF	Ni-Mn LDH MOF/GCE	Glucose	Electrocatalyst	Amperometry	0.87 μM	[175]
ZIF	Zn, Co	Co-N/Zn nanoporous carbon/GCE	Ascorbic acid	Electrocatalyst	DPV	7.65 nM	[177]
			Uric acid			0.21 nM	
ZIF-8	Pt	ZIF-8/Pt NPs/GCE	Uric acid	Distribution of Pt NPs in the porous carbon	DPV	5 μM	[178]
ZIF-67	Co, C, ZnO	Co-NCF/ZnO/GCE	Lactic acid	Electrocatalyst	Amperometry	13.7 μM	[179]
ZIF-67	ZIF-67 derived NiCo LDH, cobalt carbonate	ZIF-67 derived NiCo LDH/cobalt carbonate/CC	Glucose	Electrocatalyst	Amperometry	110 nM	[180]
ZIF-8/ZIF-67	ZIF-8/ZIF-67, Co	ZIF-8/ZIF-67/GCE	Uric acid	Electrocatalyst	DPV	0.83 μM	[176]
Zn-MOF	Zn-MOF	Ce/Zn-MOF/GCE	Uric acid	Electrocatalyst	DPV	0.51 ng/mL	[181]
Zr-MOF	ZrO_2_	ZrO_2_ porous carbon/GCE	Uric acid	Electrocatalyst	DPV	0.1 μM	[22]

BTC: benzene-1;3;5-tricarboxylic acid; BSA: bovine serum albumin; C: carbon; CC: carbon cloth; CLS: carbonized loofah sponge Co-NCF: Co-doped N-containing carbon framework; CNF: carbon nanofiber; CP: carbon nanotube/polyurethane; CPE: carbon paste electrode; LDH: layered double hydroxide; rGO: reduced graphene oxide; SWV: square wave voltammetry; TCCP: 5,10,15,20-tetrakis (4-carboxyphenyl) porphyrin; DPV: differential pulse voltammetry; CV: cyclic voltammetry.
